# Dopamine in the Brain: Hypothesizing Surfeit or Deficit Links to Reward and Addiction

**DOI:** 10.17756/jrds.2015-016

**Published:** 2015-10-23

**Authors:** Kenneth Blum, Peter K. Thanos, Marlene Oscar-Berman, Marcelo Febo, David Baron, Rajendra D. Badgaiyan, Eliot Gardner, Zsolt Demetrovics, Claudia Fahlke, Brett C. Haberstick, Kristina Dushaj, Mark S. Gold

**Affiliations:** 1 Department of Psychiatry and McKnight Brain Institute, University of Florida, College of Medicine, Gainesville, FL, USA; 2 Division of Neuroscience - Based Therapy, Summit Estate Recovery Center, Las Gatos, CA, USA; 3 Department of Psychiatry, University of Vermont, Burlington, VT, USA; 4 Department of Neurological Research, Path Foundation NY, USA; 5 Dominion Diagnostics, LLC, North Kingstown, RI, USA; 6 Division of Nutrigenomics, La Vita RDS, Salt Lake City, UT, USA; 7 Research Institute on Addictions, University of Buffalo, State University of New York, Buffalo, NY, USA; 8 Departments of Psychiatry, Neurology, and Anatomy & Neurobiology, Boston University School of Medicine, and Boston VA Healthcare System, Boston, MA, USA; 9 Departments of Psychiatry & Behavioral Sciences, Keck School of Medicine of USC, Los Angeles, CA, USA; 10 Department of Psychiatry, University of Minnesota School of Medicine, Minneapolis, MN, USA; 11 Intramural Research Program, National Institute on Drug Abuse, National Institutes of Health, Baltimore, MD, USA; 12 Eotvos Lorand University, Institute of Psychology, Department of Clinical Psychology and Addiction, Izabella utca 46., H-1064, Budapest, Hungary; 13 Department of Psychology, University of Gothenburg, Sweden; 14 Institute for Behavioral Genetics, University of Colorado Boulder, Boulder, CO, USA; 15 Department of Psychiatry, Washington University School of Medicine. St. Louis, MO, USA

**Keywords:** Dopamine receptors, Neurogenetics, Liking and wanting, Learning, Reward deficiency syndrome, Surfeit, Incentive salience

## Abstract

Recently there has been debate concerning the role of brain dopamine in reward and addiction. David Nutt and associates eloquently proposed that dopamine (DA) may be central to psycho stimulant dependence and some what important for alcohol, but not important for opiates, nicotine or even cannabis. Others have also argued that surfeit theories can explain for example cocaine seeking behavior as well as non-substance-related addictive behaviors. It seems prudent to distinguish between what constitutes “surfeit” compared to” deficit” in terms of short-term (acute) and long-term (chronic) brain reward circuitry responsivity. In an attempt to resolve controversy regarding the contributions of mesolimbic DA systems to reward, we review the three main competing explanatory categories: “liking”, “learning”, and “wanting”. They are (a) the hedonic impact -liking reward, (b) the ability to predict rewarding effects-learning and (c) the incentive salience of reward-related stimuli -wanting. In terms of acute effects, most of the evidence seems to favor the “surfeit theory”. Due to preferential dopamine release at mesolimbic-VTA-caudate-accumbens loci most drugs of abuse and Reward Deficiency Syndrome (RDS) behaviors have been linked to heightened feelings of well-being and hyperdopaminergic states.The “dopamine hypotheses” originally thought to be simple, is now believed to be quite complex and involves encoding the set point of hedonic tone, encoding attention, reward expectancy, and incentive motivation. Importantly, Willuhn et al. shows that in a self-administration paradigm, (chronic) excessive use of cocaine is caused by decreased phasic dopamine signaling in the striatum. In terms of chronic addictions, others have shown a blunted responsivity at brain reward sites with food, nicotine, and even gambling behavior. Finally, we are cognizant of the differences in dopaminergic function as addiction progresses and argue that relapse may be tied to dopamine deficiency. Vulnerability to addiction and relapse may be the result of the cumulative effects of dopaminergic and other neurotransmitter genetic variants and elevated stress levels. We therefore propose that dopamine homeostasis may be a preferred goal to combat relapse.

## Introduction

The role of dopamine (DA) in the human brain has been fraught with many theories over a 40-year span including the awarding of a Nobel Prize in 2000. Following an extensive review, Nutt et al. [1] proposed that DA may be involved in terms of psychostimulant dependence, somewhat important for alcohol, but not important for opiates, nicotine or even cannabis. We will present an alternative view based on a consensus of the literature, in favor of “Reward Deficiency” especially as a genetic trait and epigenetic effects of chronic environmental stimuli. We embrace the concept of “Reward Surfeit”, as heightened dopaminergic function, occurring on an acute basis when the craving for abusable-drugs or the aberrant behaviors occurs.

Reward Deficiency Syndrome (RDS) was coined by Blum et al. [2-3] in 1996 and indicates an insufficiency of usual feelings of satisfaction. Dysfunction in the “brain reward cascade” (BRC) results in RDS. The reward cascade describes a unique interaction between neurotransmitters (primarily serotonergic, opiodergic, GABAergic and dopaminergic). People who have a family history of alcoholism or other addictions due to specific gene variants may be born, with an inability to use or produce these neurotransmitters. Prolonged periods of stress and use of alcohol or other substances can also lead to an impairment of the brain reward cascade function [4]. When neurotransmitters are low or fail to reach the intended brain receptors, *Homo sapiens* often feel discomfort or pain. Addiction seeking behaviors resulting from a disruption of the system that normally confers satisfaction include drug and alcohol abuse, overeating, heavy cigarette smoking, gambling, and hyperactivity. Blum et al. [5] have linked these disorders to a genetic defect, especially to dysfunction of DA receptors (for example, DRD2), the genes for which show many mutant forms.

Carlsson’s group provided evidence that DA is a powerful brain neurotransmitter that controls feelings of well-being [6]. Powerful brain chemicals and neurotransmitters (e.g., serotonin and the opioids) interact with DA, each binds to specific receptors serving distinct intercellular functions that control mood and craving. The natural goal of DA homeostasis at a minimum is to induce a balance between DRD2 and DRD1 receptors leading to feelings of reward normalcy [7]. In the late 80’s it became apparent that binding of the neurotransmitter to neuronal receptors triggers a reaction that is part of the BRC [8]. There are many cases where disruption of these intercellular cascades results in aberrant RDS behaviors, including addictions, impulsivity, excessive risk taking, and even opiate reward [9]. One specific example suggests that people who have a variant in the DRD2 receptor gene (A1 form), lack a sufficient number of DA receptors in their brains to generate DA normalcy. Thus, this yields RDS, including abnormal cravings and resultant anomalous conduct. Reward Deficiency Syndrome is a complicated concept linking reward seeking with genetic and epigenetic antecedents to dopaminergic traits/states. There have been 509 articles listed in Pubmed (5-25-15) since the conceptualization of RDS in 1996.

## Reward Deficiency Syndrome and Drug Abuse

The idea that drug use is the consequence of a low functioning midbrain DA system is embedded in the reward deficiency hypothesis. Blum et al. [10] continue to posit that hypodopaminergic function predisposes an individual to seek psychoactive substances and behaviors to release DA in reward circuits of the brain to overcome DA deficits. However, Leyton [11] proposed that the specific formulation seem to reflect the now largely abandoned idea that increases in DA equal pleasure. In this posit, he references five citations that suggest that elevated DA transmission enhances the ability of stimuli that is motivationally salient to draw and sustain approach, but these behavioral effects do not derive from increased pleasure. In doing so, he referenced work from his group [12] utilizing a novel method to deplete DA called the Acute Phenylalanine/ Tyrosine Depletion method (ADTD). DA synthesis was transiently decreased in three groups of abstinent smokers (*n* = 47): (1) early low-frequency smokers, who had smoked a maximum of five cigarettes per day for less than one year, (2) stable low-frequency smokers smoking at the same level as early low-frequency smokers for at least 3 years, and (3) stable high-frequency smokers, who smoked a minimum of 10 or more cigarettes per day for at least 5 years. Motivation to obtain tobacco was measured using a progressive ratio breakpoint schedule for nicotine-containing and cigarettes without nicotine. They found that compared with a control-nutritionally balanced mixture, APTD decreased the self-administration of nicotine-containing cigarettes, in all three groups of smokers. However, motivation to use nicotine did not change.The major problem with this experiment is that it is not surprising at all that the amount of cigarette consumption decreased as DA transiently also decreased. This decrease would be expected because it is a form of psychological extinction the very basis of currently approved FDA drugs to combat cigarette consumption.

In contrast,our group has postulated that providing chronic administration of precursor amino-acids like phenylalanine / tyrosine causes a reduction in not only substance seeking but other behavioral addictions. After prolonged abstinence, people who use their drug of choice experience a powerful euphoria that often precipitates relapse. It has been postulated that this clinically observed “supersensitivity” might be tied to genetic dopaminergic polymorphisms [13]. Moreover, in individuals who carry the DRD2 A1 allele compared with DRD2 A2 allele the dopaminergic agonist bromocriptine induces stronger activation of brain reward circuitry carriers [14, 15]. Since carriers of the A1 allele have significantly lower D2 receptor density than carriers of the A2 allele, reduced sensitivity to DA agonist activity would be expected in the former. Understandably, it is perplexing that with low D2 receptor density there is an increase in reward sensitivity with the DA D2 agonist bromocriptine. A proliferation of D2 receptors has shown *in vitro* but not *in vivo* following long-term therapy with D2 agonists, such as bromocriptine. This effect of a powerful D2 receptor agonist like bromocriptine, is noteworthy because the A1 allele of the DRD2 gene is associated with increased striatal activity of L-amino acid decarboxylase, the final step in the biosynthesis of DA. This increased striatal L-amino acid decarboxylase activity appears to be a protective mechanism against low receptor density and would favor the utilization of an amino acid neurotransmitter precursor like L-phenylalanine/tyrosine for the preferential synthesis of DA.This synthesis of DA seems to lead to receptor proliferation to normal levels and results in significantly better treatment compliance only in A1 carriers [13, 16].

**Figure 1:**
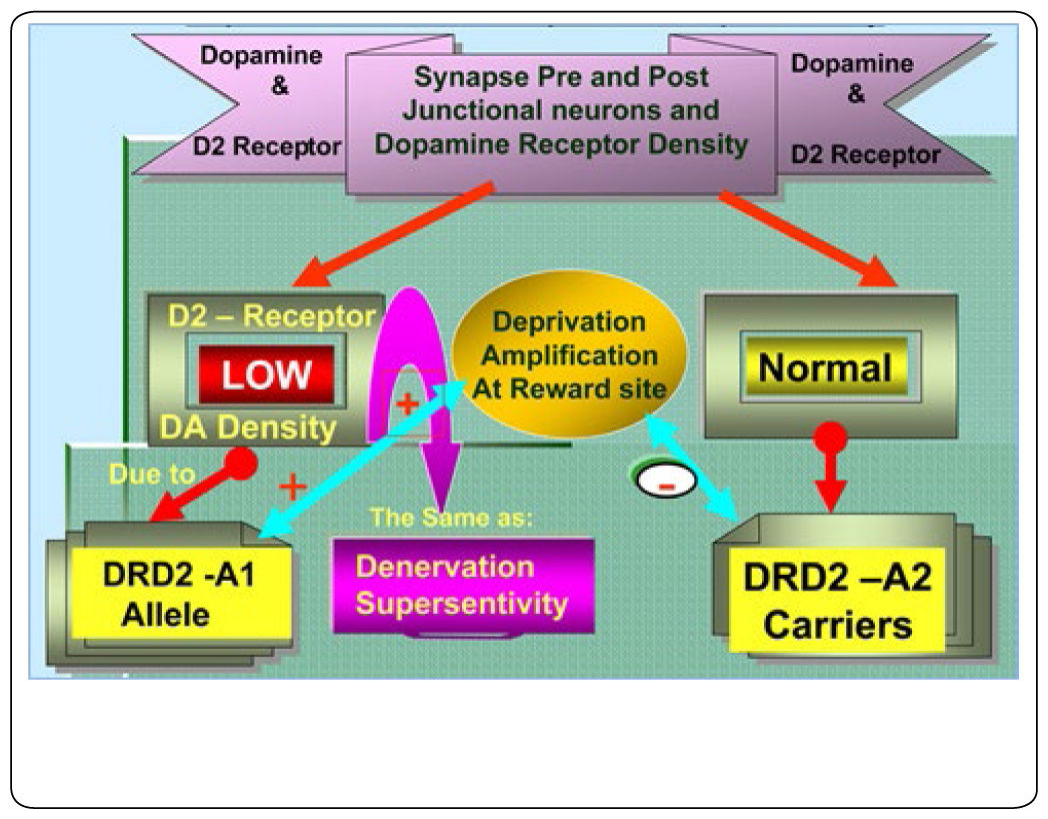
Schematic view of dopaminergic genetics and post-junction receptor density (with permission-Postgrad Med 2009 Nov; 121(6): 176–196 doi: 10.3810/ pgm.2009.11.2087)

Therefore, we propose that low D2 receptor density and polymorphisms of the D2 gene are associated with risk for relapse of substance abuse, including alcohol dependence, heroin craving, cocaine dependence, methamphetamine abuse, nicotine sensitization, and glucose craving as has been already observed by our colleagues in Sweden who demonstrated that carriers of the DRD2 A1 allele are at increased risk for drug abuse relapse [17]. “Denervation supersensitivity” is a putative physiological mechanism that may help to explain the enhanced sensitivity following intense acute DA D2 receptor activation. It was found that rats with unilateral depletions of neostriatal DA display increased sensitivity to DA agonists estimated to be 30 to 100 times in the 6-hydroxydopamine (6-OHDA) rotational model [18]. Since the DA D2 striatal receptor proliferation that occurs is mild (20%-40%), it’s hard to explain the extent of behavioral supersensitivity by a simple increase in receptor density. Alternatively, administration of a mild DA D2 agonist, would target D2 sensitization and attenuate relapse, especially in D2 receptor A1 allele carriers. This hypothesized mechanism is supported by at least 25 clinical trials which have resulted in attenuated relapse rates in reward deficiency syndrome (RDS) probands [19]. The mild DAD2 agonist utilizes amino acid neurotransmitter precursors, enkephalinase, and catechol-O-methyltransferase (COMT) enzyme inhibition. Additional translational research is required to confirm further that DA agonist therapy reduces relapse in RDS. Potentially this needed research would support the proposed concept, which we term “deprivation-amplification relapse therapy” (DART). This term couples the mechanism for relapse, which is “deprivation-amplification,” especially in DRD2 A1 allele carriers with natural D2 agonist therapy [13, 16, 20]. This supersensitivity for DA has been related to the work of Kostrzewa et al. [21] and Flagel et al. [22] that utilized two different types of animal models selectively bred based on high or low reactivity to a novel environment. In support of the DART concept [13] they found that DA D2 agonists caused greater psychomotor activation in bred high-responder rats (bHRs) relative to low-responder rats (bLRs) suggesting “dopamine supersensitivity” [21-22] (see figure 1). Moreover, relative to bLRs, bHRs had a greater proportion of DA D2 (high) receptors, the functionally active form of the receptor, in the striatum, in spite of lower D2 mRNA levels and comparable total D2 binding. Of further interest, fast-scan cyclic voltammetry revealed that bHRs had more spontaneous DA ‘release events’ in the core of the nucleus accumbens than bLRs.

Figure 1 illustrates pre- and post-junction DA neurons and the DA receptor density being low due to the DRD2A1 allele probands, compared to probands of the DRD2A2 allele with a normal compliment of D2 receptor density. This phenomenon mimics the well-known physiological mechanism “Denervation Supersensitivity”. Thus, A1 allele carriers may have “Deprivation Amplification” at the reward site in contrast to the A2 carriers, who would have a normal response to re-imbibing a psychoactive D2 agonist. It is proposed that relapse is worse for carriers of the A1 allele compared to the A2 allele of DRD2 gene.

Leyton’s group [23] also postulated that the administration of L-Dopa to so-called healthy human’s subjects did not result in any altered mood change. The authors jumped to the conclusion that DA neurotransmission does not directly influence positive mood in humans without considering many other factors including DA release. This notion has been supported by Berridge and Kringelbach [24] whereby they cite a number of neuroimaging studies indicating diverse number of pleasurable stimuli activate similar circuitry, suggesting a common neural currency shared by all. Wanting for reward is generated by a large and distributed brain system. Liking, or pleasure itself, is generated by a smaller set of hedonic hot spots within the limbic circuitry. It has been considered that opiates are more pleasure-inducing (liking) than DA (wanting) [25-27]. How then do we also explain the fact that endogenous morphine synthesis requires endogenous DA, which is thought to be necessary for endogenous brain morphine formation and even reward [9, 28]? Indeed, Charron et al. [28] found an enhanced presence of endogenous morphine in L-Dopa treated Parkinsonism. Moreover, the problem with trying to show the mood effects of L-Dopa in healthy volunteers is that the so called normal baseline of endogenous brain DA in non-genetic cases (something not characterized in Leyton’s study) and before and after L-Dopa will be very small as found by Cropley et al. [29]. The authors concluded that [18F] fDOPA is useful for measuring amphetamine-induced DA release, but may be unreliable for estimating tonic DA levels, in the striatum and extra-striatal regions of healthy humans. Importantly, even if DA’s primary role is motivation to keep using, its deficiency in the brain will have profound effects on not only relapse [13] but medical problems and severity of alcoholism [30, 31] and even mortality [32].

Along the lines of precursor amino-acids effects on patient mood, Ruhé et al. [33] provided a meta-analysis of monoamine depletion in humans. These studies included acute tryptophan depletion (ATD) or para-chlorophenylalanine (PCPA) deplete-5-HT, or acute phenylalanine/tyrosine depletion (APTD) or alpha-methyl-para-tyrosine (AMPT) deplete-NE/DA. The important message here is that depending on the population, for example, healthy controls, patients with previous major depression (MDD) in remission and patients suffering from MDD conflicting results were found. Specifically, 5-HT or NE/DA depletion did not decrease mood in healthy controls and slightly lowered mood in healthy controls with a family history of MDD. In drug-free patients with MDD in remission, a moderate mood decrease was found for ATD, without an effect of APTD. ATD induced relapse in patients with MDD in remission, who used serotonergic antidepressants. The main conclusion is that monoamine depletion studies demonstrate decreased mood in subjects with a family history of MDD and drug-free patients with MDD in remission but do not decrease mood in healthy humans. Therefore the experiment performed by Liggins et al. [23] as previously cited may be correct but their interpretation could be flawed. Other amino acid depletion work by Hildebrand et al. [34] also supports the notion that depletion of dietary L-tyrosine for example leading to lower DA resulted in an altered alertness in adult humans.

David Nutt and associates in a detailed thorough review [1] cited earlier proposed that the only robust finding showing striatal VTA DA release is for psychostimulants and alcohol with little or no evidence for opiates, nicotine, and even cannabis.

## Opiates /Opioids and Dopamine Dynamics

In response to Nutt et al. [1] a PUBMED search [5-26-15] that used the search terms was “opiates and DA release in brain” revealed at least 76 articles that went back to 1979. Deyo et al. [35] showed that various doses of morphine administered to rats resulted in an accelerated DA turnover in the neostriatum that was subsequently blocked by the opiate antagonist Naloxone. In the early 80’s Lubetzki et al. [36] showed that the endogenous opioid peptide induced DA release in rat striatum through delta opiate receptor agonism that was blocked by naloxone. In 1986, Di Chiara and Imperato [37] reported that morphine, methadone and fentanyl mu opiate agonists all stimulated DA-release and metabolism in the accumbens at lower doses than in the caudate. Specifically, maximal stimulation of DA was found with methadone at 300% above baseline in the accumbens. Interestingly, there was no increase in DA release in the brain following kappa stimulation by known kappa agonist administration. In 1991, Wood and Rao [38] showed that Morphine enhanced DA release in the rat olfactory tubercle, nucleus accumbens, prefrontal cortex and pyriform cortex, but not in nigrostriatal. The authors suggested that mesolimbic and mesocortical dopaminergic projections in the rat could be activated by opiates leading to alterations in cortical function involved in some of the complex behavioral actions of opiates and opioid peptides. In 1993, Johnson and Glick [39] used in vivo microdialysis, to compare morphine-tolerant/sensitized rats (MTSR) to controls. Basal DA concentrations and levels of DOPAC and HVA in the Nucleus Accumbens (NAc) were greater after acute morphine injection in MTSR. Bontempi and Sharp in 1997, [40] exquisitely observed that following systemic morphine administration, Fos induction in dorsomedial striatum and NAc is mediated by DA neurons in medial substantia nigra (SN) and VTA that project to medial striatum and NAc, respectively. Acute morphine administration acts on mu opioid receptors located on GABAergic interneurons in medial SN and VTA. As such inhibition of these GABA interneurons disinhibits medial SN and VTA DA neurons, producing DA release in medial striatum and NAc. In 1999, Xi and Stein [41] suggested the role of GABA in opiate-DA signaling and potentially in opiate abuse. They had reported that heroin reinforced self-administration behavior, and NAc DA release were mediated predominantly by GABA (B) receptors in the VTA. In 2005, Miller et al. [42] further suggested that midbrain DA neurons are critical in mediating the rewarding effects of opiates, as well as modulating some manifestations of opiate withdrawal in opiate dependent rats. In fact, using in vivo chronoamperometry, they found midbrain muscarinic receptors regulate morphine-induced accumbens and striatal DA efflux in the rat. It is well-known that natural reward and drugs of abuse effect mesolimbic pathways and activate common mechanisms of neural plasticity in the NAc. In 2014, Pitchers et al. [43] indicated that chronic exposure to opiates induces plasticity in dopaminergic neurons of the VTA, which regulates morphine reward tolerance. In fact, they found endogenous opioids during mating induced neural plasticity in VTA DA neurons appear to be critical for morphine reward and long-term memory for natural reward behavior.

## Nicotine and Dopamine Dynamics

In response to Nutt et al. [1], a search into PUBMED [5-26-15] revealed at least 611 articles when the search terms “nicotine and DA release in brain” was used. In 1976 Lichtensteiger et al. [44] showed that the release of DA from terminals was indicated, in caudate-putamen of rats subjected to nicotine treatment, by an increase in the DA metabolite - HVA concentration. Then in 1982 Sakurai et al. [45] reported that Nicotine, lobeline, coniine, and spartein, nicotinic agonists, significantly increased spontaneous [3H] DA release almost in a dose-dependent manner. In contrast, oxotremorine, a muscarinic agonist, did not change [3H] DA efflux. Importantly, these results suggest that n-Acetylcholine receptors on the dopaminergic nerve terminals in the striatum when stimulated by nicotine cause DA release in the brain and may have relevance to nicotine dependence. Moreover in 1985, Anderson et al. [46] in an exquisite experiment, reported that chronic cigarette smoke increased the turnover of DA in systems of the medial and lateral palisade zones. In 1989, Princeton Scientists from Hoebel’s laboratory showed that Nicotine increased extracellular DA in a dose-related manner in the nucleus accumbens [47]. They suggested that presynaptic induction of DA release might play a role in nicotine addiction. Schilstrom et al. [48] in 1998 reported that both nicotine and food-induced DA release via alpha 7 nicotinic receptors in the VTA in animal models. Rahman et al. [49] in 2003 reported that both acute and chronic nicotine exert stimulatory effects on releasing DA in the VTA. Seeking mechanisms for nicotine-induced DA release, Cheer et al. [50] in 2007, exquisitely showed that nicotine release of DA in the NAc requires activation of CB1 cannabinoid receptors. In 2011, Kleijn et al. [51] reported that nicotine directly affects an increase of DA release in NAc by local infusion. They concluded effects of nicotine on DA release at the level of the NAc might be more important for the rewarding effects than initially proposed.

Finally as recent as 2015, Justinova et al. [52] showed that the novel metabotropic glutamate receptor 2 positive allosteric modulator, AZD8529 decreases nicotine self-administration by decreasing nicotine-induced NAc DA release. This effect was also seen with food-induced DA release as well indicating shared common neuro-mechanisms.

## Cannabis and Dopamine Dynamics

The first study to evaluate the role of the effect of delta 9-tetrahydrocannabinol (THC) on the uptake and release of 14C-DA from crude striatal synaptosoma, was by Howes and Osgood [53] in 1974. Rodríguez de Fonseca et al. [54] in 1991 showed that pre and perinatal exposure to hashish exerted an important influence on both D1 and D2 DA receptors. Specifically it was found that chronic hashish increased the number of these two receptors respectively while decreasing tyrosine hydroxylase activity in the striatum. In fact, it was shown that perinatal exposure to cannabinoids like hashish, altered the normal development of nigrostriatal, mesolimbic and tuberoinfundibular dopaminergic neurons. Bossong et al. [55] employed positron emission tomography (PET) and [(11)C] raclopride the DA receptor tracer for D(2)/D(3) in healthy subjects. They were able to demonstrate that inhalation of THC reduced [(11)C] raclopride binding in the precommissural dorsal putamen and the ventral striatum but not in other striatal subregions. Reduced [(11)C] raclopride binding of is consistent with an increase in DA levels in these regions. The authors concluded that THC shares a potentially addictive property with other drugs of abuse. It suggests that the endogenous cannabinoid system is involved in regulating striatal DA release. In contrast, Urban et al. [56] using PET did not find a difference in resting state non-displaceable binding potential in abstinent mildly to moderate cannabis users compared to controls in the striatum. However, they did find an earlier age of onset abuse, correlated with lower non-displaceable binding in the associative striatum when controlling for current age, as well as, longer-term abuse.

There is some controversy related to the role of cannabis and DA release in the VTA. However, in 2012, Olson, and Cheer [57], provided a clear explanation, addressing the fact that cannabis like other drugs of abuse does indeed alter DA release in reward seeking. Accordingly, they suggest that increases in mesolimbic DA transmission are observed when animals are treated with conditioned stimuli predicting drug availability or with all known drugs of abuse, including cannabis. In contrast, decreases in mesolimbic DA function are observed during drug withdrawal, including cannabis withdrawal syndrome. They profoundly point out that despite the misconception that cannabis is unique, cannabis exerts effects on the mesolimbic DA system identical to the effects of other drugs of abuse.

Olson and Cheer point out -“recent discovery that endogenous cannabinoids modulate the mesolimbic dopamine system, however, might be exploited for the development of potential pharmacotherapies designed to treat disorders of motivation. Indeed, disrupting endocannabinoid signaling decreases drug-induced increases in dopamine release in addition to dopamine concentrations evoked by conditioned stimuli during reward seeking”.

Based on this evidence as presented above, we suggest that Nutt et al. [1] may have over simplified their view and should reconsider the role of DA in addictive behaviors. In this regard, we will review some basic established tenants related to the role of DA and reward.

## Understanding Anti Reward Mechanisms

According to Gardner [58], addictive drugs share certain attributes.They include that they are avidly and voluntarily self-administered by laboratory animals, they enhance the brain reward circuit functioning producing the ‘high’ that the drug user seek. The core reward circuitry links in-series the VTA, NAc, and ventral palladium via the medial forebrain bundle. These reward circuits are now believed to encode attention, reward expectancy, incentive motivation and hedonic tone and that hedonic dysregulation may lead to addiction [59]. In this reward circuitry, a second-stage dopaminergic component is crucial, that is the development of drug sensitivity. All addictive drugs enhance directly, indirectly or even trans-synaptically dopaminergic reward synaptic function in the NAc [60]. With chronic use of addictive drugs like cocaine or opiates tolerance to the euphoric effects develops. Post-use dysphoria then becomes the dominate hedonic tone. Addicts use drugs primarily to get back to normal and combat dysphoria. The regions of the brain responsible for inducing physical dependence for drugs may be different from those mediating pleasurable effects of addictive drugs, craving, and relapse. Variations in genetic vulnerability to drug addiction are also important, for example, variations in the DRD2 gene that encodes the DA D2 receptor. Concomitantly, vulnerability to addiction is altered by environmental factors such as stress (high stress combined with polymorphisms in dopaminergic genes, as well as other neurotransmitter genetic variants), and social defeat [61]. A bio-psycho-social model of etiology holds very well for addiction. According to Conner et al. [62], “addiction appears to correlate with a hypodopaminergic dysfunctional state within the reward circuitry of the brain, producing an addiction-prone personality”.

Neuroimaging studies in humans add credence to this hypothesis. Credible evidence also implicates serotonergic, opioid, endocannabinoid, GABAergic, and glutamatergic mechanisms in addiction as denoted in the brain reward cascade hypothesis [63]. Critically, drug addiction progresses from reward-driven to habit-driven drug-seeking behavior, escalating from occasional recreational use to impulsive use, to habitual, compulsive use.This behavioral progression correlates with a neuroanatomical progression from ventral striatal/NAc to striatal dorsal control over drug-seeking behavior. The three classical sets of craving and relapse triggers are reexposure to addictive drugs, stress, and reexposure to environmental cues (people, places, and things) previously associated with the drug-taking behavior. Drug-triggered relapse involves the NAc and the neurotransmitter DA, especially super-sensitivity of DA receptors and resting state functional connectivity [64]. Stress-triggered relapse involves the central nucleus of the amygdala, the bed nucleus of the stria terminalis, and the neurotransmitter corticotrophin-releasing factor. Stress also involves the lateral tegmental noradrenergic nuclei of the brain stem and the neurotransmitter norepinephrine. Cue-triggered relapse involves the basolateral nucleus of the amygdala, the hippocampus, and the neurotransmitter glutamate.

## What is Dopamine’s Role in “Wanting,” “Learning” and “Liking”?

While it may seem difficult to differentiate the role of DA in brain reward mechanisms, some investigators have attempted to do so. Robinson et al. [65] examined whether DA regulates liking, wanting, and/or learning about rewards during goal-directed behavior. The researchers tested genetically engineered DA-deficient (DD) mice for the acquisition of an appetitive T-maze task with and without endogenous DA signaling. They established that DD mice treated with L-dihydroxyphenylalanine (L-dopa) performed similarly to controls on a T-maze task designed to measure liking, wanting, and learning about rewards. Further experiments, however, which tested saline-, caffeine-, and L-dopa-treated DD mice on the T-maze, separated performance factors from cognitive processes, and the findings revealed that DA was not necessary for mice to like or learn about rewards, but it was necessary for mice to seek (want) rewards during goal-directed behavior. Robinson et al. [65] demonstrated that reward learning could proceed normally in the brains of DD mice, even though they contained no DA at the time of learning, if the mice were given caffeine just before learning. Caffeine activated the DD mice by an unknown non-dopaminergic mechanism, allowing them to learn where to obtain food reward in a T-maze runway. Their reward-learning-without-DA was revealed on a subsequent test day when DA function was restored by L-dopa administration. Robinson et al. [65] concluded that DA was not needed for normal learning about rewards, nor for hedonic liking of rewards during learning, but specifically for a motivational wanting component of reward-incentive salience. These results agree with the findings of Davis et al. [66] suggesting that DA is for “wanting” and opioids are for “liking”.

Wilson et al. [67] systematically explored the role of neurotransmitters in “wanting” and “liking”. They tested rats following acute, systemic administration of drugs that globally enhance serotonin and noradrenaline (imipramine), DA (GBR 12909), and opioid (morphine) function in a behavioral task designed to measure wanting and liking. Imipramine augmented the effects of delay and taste on reward “wanting”. GBR 12909 attenuated the effects of delay on reward “wanting” and the impact of taste on reward “liking,” and morphine reduced the effect of delay on a measure of reward “wanting.” Since morphine failed to effect reward “liking,” but previously had been found to enhance reward “liking” in taste reactivity tests, and since DA seemed to affect both “wanting” and “liking,” these data underscore the complexity of this concept, as well as the need for more definitive research.

However, there is evidence that DA’s function is not one of inducing pleasure per se but instead is required for seeking pleasure. The findings of Schmidt et al. [68] did not support the anhedonia hypothesis of central dopaminergic dysfunction as proposed other by investigators [69-72]. Rather, affective flattening reflected by DA receptor sensitivity may result from the lack of an effective response towards reward-indicating stimuli. These findings indicated that patients with dopaminergic dysfunction were able to experience pleasure, but may have failed to be motivated by environmental stimuli to seek reward. The complex nature of reward mechanisms is further evidenced by the work of Mirenowicz and Schultz [73]. They suggest that DA neurons in monkeys were activated by unpredicted appetitive stimuli such as food and liquid rewards and by conditioned reward-predicting stimuli. They further found that in contrast to appetitive events, primary and conditioned aversive stimuli either failed to activate DA neurons or induced weaker responses than appetitive stimuli. Thus, DA neurons preferentially reported environmental stimuli with appetitive rather than aversive motivational value.

The idea that aversive and appetitive stimuli have some similar effects is an important element of the view that DA signals salience. However, it is not only DA that behaves in this way. Peptides such as corticotropin-releasing hormone also respond similarly to both types of stimuli, although the extent of the changes is not the same. Finally, Koob and Volkow [74] in discussing the neurocircuitry of addiction emphasized the role of both impulsivity and compulsivity leading to a tripartite addiction cycle involving three stages: binge/intoxication, withdrawal/negative effect, and preoccupation/anticipation (craving). Impulsivity and compulsivity, as well as the various stages of the cycle, are tied to specific brain systems. Clearly, the picture is not a simple one.

According to a study by Sharot et al. [75], the brain chemical DA influences how people make simple and complex decisions, from what to make for dinner to whether to have children. “Humans make much more complex decisions than other animals-such as which job to take, where to vacation, whether to start a family-and we wanted to understand the role of DA in making these types of decisions.” The investigators showed that L-dopa enhanced dopaminergic function during the imaginative construction of positive future life events, subsequently enhanced estimates of the hedonic pleasure (“liking”) to be derived from these same events. These findings provided indirect evidence for the role of DA in the modulation of subjective hedonic expectations in humans.

The ponderous of evidence seems to favor the “surfeit theory” in terms of acute effects of most drugs of abuse and a number of behaviors arguing for heightened feelings of well-being linked to hyperdopaminergic states due to preferential DA release at mesolimbic- VTA-caudate-accumbens loci. Specifically, the incentive salience or “wanting” hypothesis of DA function is supported by a majority of the evidence. Neuroimaging studies have shown that drugs of abuse, palatable foods, and anticipated behaviors such as sex and gaming affect brain regions involving reward circuitry, and may not be unidirectional. Drugs of abuse enhance DA signaling and sensitize mesolimbic mechanisms that evolved to attribute incentive salience to rewards.

There is an abundance of solid research that show all addictive drugs are voluntarily self-administered, they enhance (directly or indirectly) dopaminergic synaptic function in the NAc.Addictive drugs stimulate the functioning of brain reward circuitry (producing the “high” that drug users seek). Certainly on the acute basis the responsivity of reward circuitry provides for a surfeit of DA signaling as such leads to feelings of heightened reward and reinforcement. This understanding has led to approval of FDA pharmaceuticals for alcohol, opiates and nicotine favoring blocking of DA activity with the intent of induction of extinction and such one therapeutic modality.

While we agree that dopaminergic antagonistic therapy may be useful in the short-term, we argue against its use in the long term. Our argument recognizes that “dopamine hypotheses” originally thought to be simple, now is believed to be quite complex. Encoding the set point of hedonic tone, encoding attention, reward expectancy, and incentive motivation are all involved.

Most importantly Willuhn et al. [76] show that in a self-administration paradigm, (chronic) excessive use of cocaine results from decreased phasic DA signaling in the striatum. This work supports the notion that long–term DA agonistic therapy may be more prudent and may indeed be a new addiction modality. Finally, being cognizant of the differences between dopaminergic function, we argue that relapse may be tied to DA deficiency as found in carriers of the DRD2 A1 allele and as such require agonistic rather than antagonistic therapy. Elevated stress levels, together with polymorphisms of dopaminergic genes and other neurotransmitter genetic variants, may have a cumulative effect on vulnerability to addiction. The RDS model of etiology holds very well for a variety of chemical and behavioral addictions.

## Reconsideration

The initial RDS hypothesis suggested that a dysregulation or dysfunction of mesolimbic DA induces a motivation for seeking reward based stimuli [2-3]. Substantial subsequent evidence accrued to show that a driving force for drug use was ‘liking’ and not just ‘wanting’ [77], but some evidence also showed DA has a role of ‘learning’ [78]. Based on the accumulation of evidence, we recommend that RDS should now be redefined to specify the distinct role of DA for “wanting,” “learning,” or “liking”. However, the RDS hypothesis continues to posit that hypodopaminergic function predisposes an individual to seek psychoactive substances and behaviors to release DA in reward circuits of the brain to overcome DA deficits. Hedonic dysregulation within these circuits may lead to addiction [79].

The second-stage dopaminergic component in this reward circuitry is the crucial addictive-drug-sensitive component. All addictive drugs have in common that they enhance (directly or indirectly or even trans-synaptically) dopaminergic reward synaptic function in the NAc [37]. Drug self-administration is regulated by NAc DA levels and is done to keep NAc DA within a particular elevated range (to maintain a desired hedonic level). Importantly bear in mind that an older DA hypothesis [80], a single system model, posited that the neurotransmitter DA played a fundamental role in mediating the rewarding properties of all classes of stimuli.

In contrast, both non-deprived/deprived, and saliency attribution models claim that separate systems make independent contributions to reward. The former identifies the psychological boundary defined by the two systems as being between states of non-deprivation (for example, food sated) and deprivation (for example, hunger). The latter identifies a boundary between liking and wanting systems. In doing so, the newer understanding by Berridge and others [1, 81] even involving food addiction does not negate the underlying cause of addiction as proposed by the RDS concept.

While there is still controversy concerning the role of certain drugs of abuse and dopamine activation during different phases of abuse –acute vs chronic, Sami et al. [82] pointed out in a recent meta-analysis that the modulation effect of THC on DA neurotransmission was a function of the time of utilization of Cannabis and type of subjects studied. It appears that in healthy male subjects the acute effects of THC may not be striking, but the chronic effects do show significant DA blunting. Moreover, males that have a high genetic risk for psychosis do show that acute cannabis exposure increases dopamine release in striatal and pre-frontal areas. With this information it would be of interest to determine if the same is true for carriers of polymorphic genes that increase risk for RDS.

In this regard, van der Knaap et al. [83] found that adolescents with the Met/Met genotype and high rates of Membrane Bound (MB) -COMT promoter methylation were less likely to be high-frequent cannabis users than adolescents with the Val/Val or Val/Met genotype. The authors suggest although these results are complex they indicate that there is an association between substance use and COMT gene methylation. In addition, Szutorisz et al. [84] showed that parental THC exposure leads to compulsive heroin –seeking as well as changes in the mRNA expression of cannabinoid, dopamine, and glutamatergic receptor genes in the striatum (altered striatal synaptic plasticity) in the subsequent generation. DiNeri et al. [85] similarly found that maternal cannabis use alters ventral striatal dopamine D2 receptor gene regulation in the offspring. Specifically, decreased DRD2 expression was accompanied by reduced dopamine D2 receptor (D(2)R) binding sites and increased sensitivity to opiate reward in adulthood.

Finally there is even evidence that selected polymorphisms of the dopamine receptor gene DRD2 and the ANKK-1 impact preference for sucrose solutions. In fact, Jabłoński et al. [86] showed that the Taq-1A polymorphism plays a role in the preference to high concentrations of sucrose and its potential association with alcohol dependence pathogenesis. Indicating that dopamine plays a role in food addiction co-morbidly with other similar drug addictions [87].

## Conclusion

In our view, the role of DA deficiency remains key in reward-seeking behavior and motivation to continue its seeking. Further research using imaging tools and genetics and epigenetics will provide important adjunctive information necessary to fully characterize the role of DA in reward circuitry and RDS behavior. Are we throwing out the baby with the bathwater?
